# Challenges of Providing Access to Cutting-Edge Cancer Medicines in the Countries of Eastern Europe

**DOI:** 10.3389/fpubh.2018.00193

**Published:** 2018-07-24

**Authors:** Zdenko Tomić, Ana Tomas, Zuzana Benšova, Ljiljana Tomić, Olga Horvat, Ivan Varga, Milica Paut Kusturica, Ana Sabo

**Affiliations:** ^1^Department of Pharmacology and Toxicology, Faculty of Medicine Novi Sad, University of Novi Sad, Novi Sad, Serbia; ^2^Department of Gynecology, Clinical Centre of Vojvodina, Novi Sad, Serbia; ^3^University of Bijeljina, Dvorovi, Bosnia and Herzegovina; ^4^Institute of Radiology, Institute for Child and Youth Health Care, Vojvodina, Novi Sad, Serbia

**Keywords:** targeted therapy, monoclonal antibodies, protein kinase inhibitors, HTA, balkans

## Introduction

Cancer is a leading cause of death (1)-based on WHO data, 8.2 million people die each year from cancer, an estimated 13% of all deaths worldwide (2). Cancer medicine is an important component of overall health care costs (3, 4). The shift from conventional cytotoxic drugs to targeted cancer therapies (TCT) has caused the cost for cancer treatment to rise substantially with often modest survival benefits and sometimes only for the purpose of palliative treatment (5–8). There is currently little association between therapeutic effects and requested prices, e.g., of the 12 drugs approved by the FDA for cancer in 2012, 9 were priced at more than US$10,000/month with only 3 prolonging survival, two by less than 2 months (5, 9). Drug expenditure is one of the largest components of health spending in Eastern Europe (10, 11), i.e., spending for monoclonal antibodies used in malignancies in Serbia has shown 20-fold increase from 2004 to 2012 (7). Several studies that compared availability and reimbursement of TCTs among different health care settings, regions and countries, demonstrated high rate of variation in number of medications reimbursed between EU and USA (12–16), as well as among different EU countries (16–20). With large discrepancy between cost and clinical benefit for some TCTs (6, 18, 21), reimbursement of these medication represents an issue even for high income countries. There is limited data about the use and reimbursement of these drugs in former Yugoslavia (22). Countries of former Yugoslavia deal with limited financial resources available for health care spending, based on payroll taxation as a major source of financing and most public expenditure on health flows through the health insurance funds (23). High unemployment rates, and a large share of the active labor force working in the informal sector where contributions to health insurance are not made (22, 24), further complicates the functioning of insurance health funds. Countries of former Yugoslavia, being in the process of harmonization with the EU, try to bridge the gap between the conflicting need to reimburse novel, expensive medication and lack of resources (25, 26).

## Financial burden of drugs for malignancies

The cost of delivering high quality cancer care is outstripping the national budgets of countries of former Yugoslavia. Serbia (SRB), Bosnia and Herzegovina (BiH), FYR Macedonia (MKD) and Croatia (HRV) are classified as upper middle income, while Slovenia (SVN) is the only high income country according to the World Bank data. As witnessed by current estimates given in Table 1, health care financing remains under pressure—Serbia and Republic of Srpska (RS) spend the largest share of GDP on healthcare, but actual health expenditure per capita is only around 500 USD. Pharmaceutical expenditure is one of the key components in increased health-care spending, and cancer treatment is important component of pharmaceutical expenditure. Between 10 and 25% of total pharmaceutical expenditure in eastern Europe was related to the procurement of drugs for malignancies. For referencing, Slovakia (SVK), an EU country which shares large number of similarities to the countries of the former Yugoslavia region^1^, spent almost a quarter of total pharmaceutical expenditure on drugs for malignancies. Aside from the other health costs associated with cancer, pharmacotherapy is an important component of the cancer treatment—recent study conducted in Serbia concluded that pharmacotherapy costs accounted for 42.37% of all cancer related health care expenditure (27). TCTs are the major cancer care cost drivers in Eastern Europe as these drugs are relatively expensive in comparison to the other cancer treatment options. Monoclonal antibodies (Table 2) and protein-kinase inhibitors (Table 3) absolute expenditure is much lower in RS and Serbia compared to Slovakia, Slovenia, and Croatia, but high proportion of pharmaceutical spending stems from TCT procurement. Given the increase in malignancy incidence, aging population, and lack of fully implemented screening programs, the financial pressure novel cancer treatments pose on budgets in countries in transition is expected to increase further in the future. Differences in TCTs spending might be related to differences in level of reimbursement across Eastern Europe, as these large expenses are limiting factor for reimbursement of these medication (26), especially in low resource settings.

**Table 1:** Characteristics of countries: basic health care parameters^a^, health spending^b^ and spending for drugs for malignancie^c^.

	**SVN**	**SRB**	**BiH/ RS**	**HRV**	**MKD**	**MNE**	**SVK**
Population (millions)	2.06	7.14	2.37/1.3	4.24	2.08	0.63	5.4
GDP per capita (USD)	24020.7	6152.9	4851.7	13475.2	5469.2	7378.3	18500.7
Health expenditure as a share of GDP (%)	9.2%	10.6%	9.6%	7.8%	6.5%	6.4%	8.1%
Health expenditure per capita (USD)	2161	633	464	1050	354	458	1455
Spending for drugs for malignancy in 2014 (euro/1000inhabitants)	39447.7	12350.8	8385.1/ 5198.6	29476.7	NA	17385.7	56649.6
Spending for drugs for malignancy in 2014 (as a share of total drug spending)	18.6%	18.9%	11.3%/ 11.3%	18.7%	NA	19.4%	24.5%
Spending for drugs for malignancy (order of group L in relation to other ATC groups) in 2014	1st	4th	4th/5th	1st	NA	1st	1st

a *WHO database*,

b *World Bank database*,

c *SRB, BIH, SVN, MKD, HRV - annual reports available on the websites of state drug agencies and health funds; SVK - MCR Spotreba software; RS and MNE – data provided directly by the respective Health Funds*.

**Table 2:** Pharmaceutical spending for drugs from group L01XC (monoclonal antibodies) in euro/1000inhabitants.

**ATC INN**	**Slovenia**	**Serbia**	**Croatia**	**BiH**	**Republic of Srpska**	**Montenegro**	**Slovakia**
L01XC02 rituximab	3691.3 (14.9%)	822.8 (14.1%)	1699.9 (12.3%)	655.3 (12.9%)	861.1 (29.1%)	1529.3 (8.8%)	1791.7 (8.4%)
L01XC03 trastuzumab	2335.3 (9.4%)	1794.9 (30.9%)	2635.6 (19.0%)	1891.9 (37.4%)	1572.6 (53.2%)	1844.5 (10.6%)	2409.8 (11.2%)
L01XC06 cetuximab	717.0 (2.9%)	261.7(4.5%)	140.4 (1.0%)	509.2 (10.1%)		249.4 (1.4%)	934.9 (4.4%)
L01XC07 bevacizumab	3483.9 (14.1%)	157.7(2.7%)	1514.8(10.9%)			1482.8 (8.5%)	4940.7 (23.0%)
L01XC08 panitumumab	411.2 (1.6%)			13.2 (0.3%)			513.5 (2.4%)
L01XC13 pertuzumab		3.7(0.1%)	5.(0.0%)				3.0 (0.0%)
other	988.2 (4.1%)		96.0 (0.7%)				
L01XC total	11626.9 (54.1%)	3040.8 (52.3%)	6091.9 (44.0%)	3069.6 (60.6%)	2433.7 (82.3%)	5105.9 (29.3%)	10062.7 (49.4%)

*Data sources: SRB, BIH, SVN, MKD, HRV - annual reports available on the websites of state drug agencies and health funds; SVK - MCR Spotreba software; RS and MNE – data provided directly by the respective Health Funds. Percentages represent share of L01X group*.

**Table 3:** Pharmaceutical spending for drugs from group L01XE (TK inhibitors) in euro/1000inhabitants.

**ATC – INN**	**Slovakia**	**Croatia**	**Republic of Srpska**	**BiH**	**Montenegro**	**Serbia**
L01XE01 imatinib	1856.4(8.7%)	1594.3(11.5%)	309.0 (10.4%)	271.8(5.4%)	1262.2(7.3%)	741.2(12.7%)
L01XE02 gefitinib	746.7(3.5%)	20.5(0.1%)			26.5(0.2%)	0.0(0.0%)
L01XE03 erlotinib	1047.1(4.9%)	482.2(3.5%)		247.7(4.9%)	1098.1(6.3%)	85.9(1.5%)
L01XE04 sunitinib	1225.6(5.7%)	1242.5(9.0%)		254.9(5.0%)	641.2(3.7%)	437.5(7.5%)
L01XE05 sorafenib	470.5(2.2%)	161.1(1.2%)		285.0(5.6%)	182.5(1.1%)	2.2(0.0%)
L01XE06 dasatinib	353.2(1.6%)	298.2(2.2%)			129.0(0.7%)	
L01XE07 lapatinib	445.2(2.1%)	138.6(1.0%)		12.5(0.2%)	102.0(0.6%)	60.7(1.0%)
L01XE08 nilotinib	969.3(4.6%)	1181.5(8.5%)		693.2(13.7%)	468.8(2.7%)	322.9(5.6%)
L01XE09 temsirolimus		29.7(0.2%)				
L01XE10 everolimus	772.6(3.6%)	220.9(1.6%)			284.3(1.6%)	11.3(0.2%)
L01XE11 pazopanib	934.0(4.4%)	70.5(0.5%)			31.8(0.2%)	6.8(0.1%)
L01XE15 vemurafenib		240.2(1.7%)			224.2(1.3%)	23.6(0.4%)
Other^*^	241.4(1.6%)	13.9(0.1%)		123.3 (2.44%)		
L01XE total	9062.0(42.8%)	5694.1(41.1%)	309.0(10.4%)	1888.4 (37.2%)	4450.4(25.6%)	1692.1(29.1%)

* *Others include: in Slovakia crizotinib (45.7,0.7%), axitinib (55.7, 0.3%), ruxulotinib (117.5,0.5%), regorafenib (22.5,0.1%); in Croatia ruxulotininb (13.9, 0.1%). No data available for Slovenia*.

## Reimbursement of novel drugs for malignancies

Where there are specialized national heath technology assessment (HTA) frameworks present, the process of reimbursement of novel cancer treatments is based on pharmacoeconomic evaluation which is necessary in order to maximize the cost-effectiveness of these expensive medication (25, 28). Within EU, there are large differences in the number of approved TCTs and stringency of levels at which drug is considered cost-effective (29). Also, some of the TCTSs are subjected to special evaluations as they are considered orphan drugs. The EU has created a common procedural framework through the adoption of Transparency Directive (Council Directive 89/105/EEC) to ensure that national pricing and reimbursement decisions are made in a transparent manner. In the Balkans, such information is not readily available. Each country uses different schemes and policies for the pharmaceutical pricing and reimbursement adapted to its own economic and health needs (11, 28, 30, 31). Positive drug lists are available on the websites of the respective health funds, but the information about the assessment guiding the reimbursement decision process is difficult to find. Cost-effectiveness studies on TCT in oncology have been scarcely reported in published literature in the Balkan region (32).

**Figure 1:**
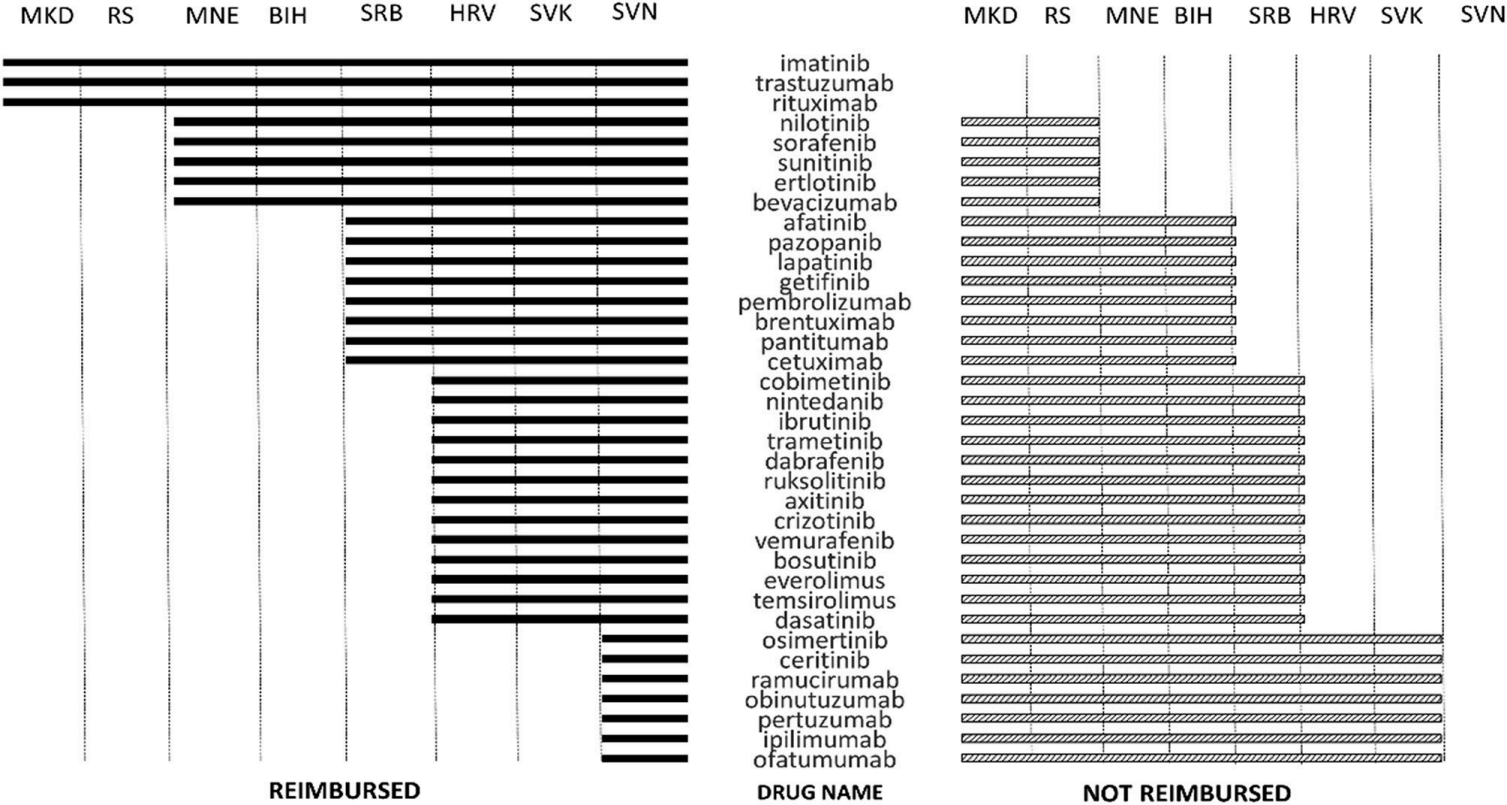
Reimbursement status of innovative oncological medicines in countries of former Yugoslavia on September 1st, 2017. Source websites of the respective websites with the last available positive drug list on the date of data extraction.

Eastern Europe belongs to the quite a different healthcare milieu compared to the developed Western economies (25). Following the fall of the SFRJ, newly formed countries struggled with remnants of socialistic health policies and the rising expenses of modern healthcare. The systems in place could no longer meet the needs of growing and aging population. While many of the Balkan countries nominally guarantee universal healthcare coverage through compulsory health insurance (26) in reality there are numerous obstacles in providing health care services in the former Yugoslavia region. Countries with lower GDP such as RS, Montenegro, Macedonia, and Serbia, all have good access to classic cytotoxic drugs, but availability of novel cancer therapies is limited. As of 1st September 2017, only rituximab, trastuzumab, and imatinib are reimbursed in all of the countries of former Yugoslavia (Figure 1). However, drugs such as rituximab, trastuzumab, and imatinib, drugs included on the WHO list of essential medicines and considered standard of care for a range of malignancies are reimbursed in all surveyed countries. However, more developed countries such as Slovenia and Croatia have higher number of reimbursed TCTs than other countries of former Yugoslavia. These differences might be related to different mechanisms of reimbursement decision making. In Bosnia and Herzegovina, health decision processes are carried out by the expert boards of the health funds (33). In the RS, drugs for malignancies are listed on the separate list of cytotoxic drugs. The reimbursement decisions are based on the experience of Serbian tertiary health care institutions and recommendations of the European Society for Medical Oncology. In the Federation of BiH, drugs for malignancies are included on the special positive list, so called “solidarity list” which includes drugs for malignancies, multiple sclerosis, hemophilia, HIV and other diseases. Solidarity fund aims to provide universal coverage of the patients with specific condition throughout the 10 cantons in BiH federation. Small number of registered innovative and brand medicines in Macedonia is a result of the generic prescribing policies, the delays, and the strict inclusion rules for the positive list (34). Some novel medicines have been rejected on the justification for limited national financial means and the existence of therapeutic alternatives. Serbia made effort to increase the availability of cutting edge cancer treatment, and in the 2016 revision of positive list included large number of TCTs not formerly available, making the reimbursement status similar to Croatia and Slovenia. The impact of this change on patient outcomes and expenditure need to be assessed in the future. There is no national HTA agency in Serbia, but the National Health Insurance Fund is involved in pharmacoeconomic assessment and health ministry includes an HTA Committee to support reimbursement decisions. As mentioned before, no information about pharmacoeonomic assessment guiding the reimbursement decisions can be found on the respective websites. This is the consequence of the fact that comprehensive and consistent systems for HTA are non-existent or underdeveloped in most of the region (33, 35). Despite some progress, pharmaceutical market is still inadequately responsive to population needs (8). On the contrary, Slovakia has national HTA agency, and assessment of added therapeutic value in Slovakia is conducted during the decision making process on the reimbursement of medicines. The pharmacoeconomic analysis is conducted by a specialist working group and these reports are mandatory during the reimbursement process (28). Out of countries of former Yugoslavia, only Slovenia and Croatia have made steps toward full HTA implementation (3, 30, 36, 37). In 2006, Croatia developed strategy for the development of the Croatian health care system which included formation of the independent, non-profit institution called Agency for Quality and Accreditation in Health. This is the basis for the HTA procedures in Croatia. Furthermore, Croatia has a special Fund for very expensive drugs 400.000.000,00 HRK (53 million €) (38). In Slovenia, HTA-related processes are carried out by the Agency for medicinal products and medical devices of the Republic of Slovenia (JAZMP) and National Institute of Public Health (NIJZ) and autonomous research organizations (39). No national HTA organization has been established, but the results of HTAs have to be considered in decision making process for planning, budgeting, pricing and reimbursement of health products. However, it is still not obligatory for the conclusion and policy outcomes of the HTA to be publicly available. In other countries, currently there is no formal HTA system in place (40). Deficiency of a HTA system and therefore inefficient procurement processes mean that the Balkan countries often invest in less cost-effective medicines and sometimes pay more than west European countries (3, 11, 26, 28). Several of the monoclonal antibodies were deemed not cost-effective for all of the recommended indications according to the NICE National Institute for Health and Care Excellence (NICE), one of the most important HTA agencies in Europe, criteria (11). Technologies not cost-effective by NICE appraisal are probably not going to be worth the resources in current setting. In order to make the most of the funds available, countries of former Yugoslavia need to develop mechanisms for development and implementation of HTA systems in the drug reimbursement processes. These strategies could help with overcoming difficulties in funding and delivering medical care in emerging markets with a rapidly growing demand for health services (23).

## Implications

Large variations and inconsistencies in the decision and processes of assessing and determining the reimbursement of novel cancer treatments was noted in the present study. This extent of the regional disparities in availabilty of TCTs should motivate the decision makers in the region to identify and implement innovative financing mechanisms to expand financial resources available for reimburesemt of novel cancer medication. National systems need to be regularly reviewed and adapted in order to take into account market evolutions and patients' needs. Comprehensive system rooted in responsible reimbursement policy based on cost–effectiveness principles is needed for assessing both new and existing healthcare technologies. The optimum strategy to achieve value in the provision of cancer care in the Balkans needs to be developed.

## Author contributions

AS and ZT Study conception and design. LT, IV, and ZB Acquisition of data. AT, OH, and MPK Analysis and interpretation of data. AT and ZB Drafting of manuscript. AS and ZT Critical revision.

### Conflict of interest statement

The authors declare that the research was conducted in the absence of any commercial or financial relationships that could be construed as a potential conflict of interest.

## Footnotes

^1^Historical background, healthcare system with compulsory public health insurance as a main pillar of health financing, largely centralized health-care system with the overall power trusted to the ministries of health and social welfare and the health insurance funds and remnants of the old system based on the principle of free treatment with the proportion of health funding from private insurance or direct payments still marginal in comparison to the western Europe average.
